# Exploration of the Experience of Care Home Managers of COVID-19 Vaccination Programme Implementation and Uptake by Residents and Staff in Care Homes in Northern Ireland

**DOI:** 10.3390/vaccines9101160

**Published:** 2021-10-10

**Authors:** Linda Craig, Radi Haloub, Heather Reid, Dalrene Masson, Hannah Mccalmont, Kathy Fodey, Barbara R. Conway, William J. Lattyak, Elizabeth A. Lattyak, Amie Bain, Sayer Al-Azzam, Mamoon A. Aldeyab

**Affiliations:** 1 Public Health Agency, Belfast BT2 8BS, UK; linda.craig3@hscni.net (L.C.); heather.reid@hscni.net (H.R.); dalrene.masson2@hscni.net (D.M.); hannah.mccalmont@hscni.net (H.M.); kathy.fodey@hscni.net (K.F.); 2 Department of Management, University of Huddersfield Business School, Huddersfield HD1 3DH, UK; r.haloub@hud.ac.uk; 3 Department of Pharmacy, School of Applied Sciences, University of Huddersfield, Huddersfield HD1 3DH, UK; b.r.conway@hud.ac.uk; 4 Institute of Skin Integrity and Infection Prevention, University of Huddersfield, Huddersfield HD1 3DH, UK; 5 Scientific Computing Associates Corp, River Forest, IL 60305, USA; blattyak@scausa.com (W.J.L.); elizabeth.lattyak@sbcglobal.net (E.A.L.); 6 Wye Valley NHS Trust, Hereford County Hospital, Hereford HR1 2ER, UK; amie.bain@nhs.net; 7 Department of Clinical Pharmacy, Faculty of Pharmacy, Jordan University of Science and Technology, Irbid 22110, Jordan; salazzam@just.edu.jo

**Keywords:** care homes, managers, COVID-19 vaccine, staff, residents, vaccine uptake

## Abstract

The Coronavirus 2019 (COVID-19) pandemic disproportionately affected people living and working in care homes. This study aimed to explore the experience of care home managers on the implementation and uptake of the COVID-19 vaccination programme by residents and staff in care homes in Northern Ireland. An exploratory mixed methods approach was used, i.e., semi-structured interviews to design the cross-sectional survey and content analysis of statements using open ended questions. Care home managers were approached and sixty-seven valid quantitative and forty-nine descriptive responses were analysed. The study identified eight themes which described factors that motivated residents (family visits and relationship with managers and staff), and staff vaccine uptake (return to normal life at work and trust in care home managers). The identified themes also confirmed that vaccine uptake is negatively influenced by perceived side effects. The findings indicated that social media can promote or decelerate the uptake of vaccine despite the accessibility to a successful vaccination programme. The study highlights the important role of managers in handling the challenges through building trust and establishing relationships with staff and residents. The findings identified challenges to the uptake of the COVID-19 vaccine by staff and residents that can inform the implementation of future vaccination programmes.

## 1. Introduction

Since the emergence of the coronavirus 2019 (COVID-19) pandemic, people living and working in care homes have been disproportionately affected [1,2,3]. Care home residents have a greater risk of suffering from severe COVID-19 infections and high rates of mortality due to older age, frailty, disability, and multiple long-term medical conditions [2,4,5,6,7,8,9]. In addition, residents in care homes live in close accommodations and have frequent contact with staff, increasing the risk of disease transmission and outbreaks [10,11,12]. Managers and care home owners are responsible through planning and staff training for ensuring that care homes offer a safe working environment, can provide care for those most at risk from COVID-19 and minimize the risk of transferring infections [13].

Vaccines have enabled the public to hope for a potential return to a more normal life despite the COVID-19 pandemic [14,15]. Given the increased risk of outbreaks, morbidity, and mortality in the care home setting, the UK Joint Committee on Vaccination and Immunization (JCVI) advised that care home residents should be the highest priority for vaccination [16], and that vaccination of staff at the same time would offer a highly efficient strategy [15]. However, successful implementation of a vaccine programme in the care home sector is dependent on successful resident and staff uptake of approved vaccines. In Northern Ireland (NI), vaccination of care home residents and staff started on the 8^th^ December 2020. The Pfizer vaccine was deployed, and the dose interval was 21 days, except in cases where the vaccination team could not visit due to any disease outbreak. A pilot study in four care homes in NI showed variations in vaccine uptake, for both the first and second doses, amongst residents and staff, warranting further assessment on a larger scale [17].

Several studies have been published with the aim of understanding population concerns and characteristics associated with lower levels of vaccine uptake and promotion [18,19,20,21,22,23]. Recently, healthcare workers’ perceptions and attitudes towards the UK’s COVID-19 vaccination programme were investigated using qualitative research [24], and the results demonstrated that vaccine-related misinformation impacted confidence and trust in the vaccination programme. Moreover, some staff at care homes were shown to be reluctant to be vaccinated if they are not directly involved with COVID-19 patients [25]. This puts pressure on the managers of care homes to convince staff and residents to receive the vaccine. Studies to understand the experiences of care home managers on COVID-19 vaccination programmes are lacking in the literature. There is a paucity of research that explores how managers perceive and identify important challenges. Using an exploratory mixed method approach, this study aimed to explore the experience of care home managers regarding the implementation and uptake of the COVID-19 vaccination programme by residents and staff in care homes in Northern Ireland (NI).

## 2. Materials and Methods

The target population for this study was managers of public and private care homes in NI (*n* = 471). To address the aim of the research, we followed an embedded design as a single data set would not be sufficient [26], and used mixed methods at three different stages. In Stage One, we commenced semi-structured interviews with care home managers to identify core themes within the experience and identify the main challenges regarding vaccine uptake by residents and staff. Data were coded by one researcher and checked by another researcher. The themes were identified that subsequently informed the questions used in the questionnaire stage (Stage Two). In Stage Two we developed a questionnaire (Questionnaire S1—Supplementary Materials) that was administered as a survey to care home managers via the Public Health Agency (PHA). Five open-ended questions were added to the questionnaire to gain further insights into the challenges that care home managers face. In Stage Three content analysis was used to interpret the descriptive answers to the five open-ended survey questions in Stage Two [27,28]. We inductively classified the codes and identified patterns in the statements provided by respondents. Themes were identified through inductive reasoning [29]. The data collection was completed by July 2021.

### 2.1. Stage One: Data Collection and Analysis of Semi-Structured Interviews 

Based on the study objectives, a semi-structured guide was developed with a variety of open-ended questions as guidance for each interview session (Semi-structured guide S1—Supplementary Materials). Consent to participate in the interviews was obtained from each participant prior to the commencement of interviews (Consent form S1—Supplementary Materials). Interviews were conducted with seven care home managers through individual telephone calls and the Zoom online platform (a total of 188 min). All sessions were transcribed verbatim by one researcher from the study team. The analysis involved coding iterations to generate key themes using thematic analysis (Table S1, Figure S1—Supplementary Materials). NVivo software (QSR International—NVivo 20 (R1)) was used to code and facilitate analysis of the data.

### 2.2. Stage Two: Data Collection and Analysis of Quantitative Data

Based on the analysis of the semi-structured interviews, described in Stage One, the identified codes and themes were used to inform the development of a questionnaire (Questionnaire S1—Supplementary Materials).

A cross-sectional design was adopted, and the survey was administered online to care home managers. A web-based survey, using Qualtrics Platform (Qualtrics, Provo, UT, USA. https://www.qualtrics.com), was used to collect and generate data. A link and invitation letter (Invitation letter S1—Supplementary Materials) were sent to participants via email by PHA (NI). After three weeks, a reminder email and invitation letter were sent (Invitation letter S1—Supplementary Materials). Participants were informed that all collected information was anonymised and no personal data or identifiers were recorded or stored. Managers were also informed that by completing this survey, their consent to participation on this study was implied. The 20 min online questionnaire was tested for content and face validity by experts in the field who provided constructive feedback. Pilot testing was also conducted with care home managers, and these data were not included in the final sample.

The survey consists of five sections comprising a total of 61 questions. The first section refers to general information about the care home to support analysis of the experience according to context. The second and third sections refer to the uptake of the COVID-19 vaccination programme in care homes by residents and staff respectively. The fourth section refers to the impact of social media and source of information on the uptake of the COVID-19 vaccination programme in care homes by staff and residents. The fifth section refers to the overall impact and implementation of the COVID-19 vaccination programme at the care homes.

There are 471 care homes in NI that define our eligible target population. Due to the small eligible population size, the minimum sample size was computed using a normal approximation to the hypergeometric distribution. With 67 survey responses from the 471 care home target population, the maximum margin of error for this survey, MOE90 (0.50), is 9.35%

The following hypotheses were tested:

**H_1_:**  *There is a relationship between the motivation for vaccine uptake and connotation (positive or negative) messages on social media at care homes in NI.*

**H_1.1_:**  *There is a relationship between the motivation of staff for vaccine and messaging uptake (positive or negative) on social media at care homes in NI.*

**H_1.2_:**  *There is a relationship between the motivation of residents for vaccine and messaging uptake (positive or negative) on social media at care homes in NI.*

**H_2_:**  *There is a negative relationship between the motivation for vaccine uptake and the perceived safety of the vaccine at care homes in NI.*

**H_2.1_:**  *There is a negative relationship between the motivation for staff vaccine uptake and the perceived safety of the vaccine at care homes in NI.*

**H_2.2_:**  *There is a negative relationship between the motivation for residents’ vaccine uptake and the perceived safety of the vaccine at care homes in NI.*

**H_3_:**  *There is a relationship between the motivation for vaccine uptake and the process of implementation of the vaccination programme in care homes in NI.*

Reference to the questions that were used to test the above hypotheses are presented in Table S2. The actual questions are presented in Questionnaire S1—Supplementary Materials.

Cronbach’s alpha tests were used on the individual sections of the survey to assess internal consistency (Table 1). All values suggest reasonable reliability and therefore measure the same concept or construct.

The Likert scale (1 = Strongly disagree to 6 = Strongly agree) used in the survey translates into measuring agreement to the questions. As part of the analysis, the means of the various questions were computed, along with their 95% confidence limits. Means that approach 6 indicate a consensus of strong agreement to the question, whereas means that approach 1 indicate consensus of strong disagreement. The confidence limits were bootstrapped instead of using the standard deviation of the question responses.

A one-sample Wilcoxon test was used to compare the means of the question responses to be less than, or greater than, key ordinal values of the Likert scale. Specifically, we are interested in knowing if the probability of the mean response is below 3 (somewhat disagree), or whether the mean is greater than or equal to 4 (somewhat agree).

We also determined a Net Agreement Score (NAS) computed by subtracting the percentage of question responses that scored 3 or less (somewhat disagree to strongly disagree) from the percentage of question responses that scored 4 or more (somewhat agree to strongly agree). This generated is a score between −100 and +100 which is a distributional measure of agreement to the question. An NAS score of +100 indicates 100% agreement to the question while an NAS score of −100 indicates 100% disagreement to the question. An NAS score of 0 indicates an equal number of agreement and disagreement to the question.

Prior to the survey analysis, the survey questions were grouped into the following constructs (or categories): motivation for vaccine uptake by residents; motivation for vaccine uptake by staff; social media influences on residents; social media influences on staff; safety influences on residents; safety influences on staff; implementation influences on residents; and implementation influences on staff.

Pearson correlation tests were employed to examine the linear relationship between questions in each topic group (social media, safety, implementation) versus the questions associated with vaccine uptake motivation. This information was used by the authors to explore micro-level relationships of drivers for uptake motivation.

A factor analysis was performed on the grouped questions for each topical construct. We sought to create a single factor for each construct (or category) using principal component analysis (PCA), exploratory factor analysis (EFA), and confirmatory factor analysis (CFA) methods. The ordinal scores of negative phrased questions were reversed prior to applying a PCA/factor analysis on the grouped questions so that agreement was always associated with increased uptake motivation.

The factor analysis on social media for staff produced both significant positive and negative factor loadings caused by mixed phrasing of the questions in the construct. The questions were separated by positive and negative phrasing and a factor analysis for this topic and a factor analysis was conducted separately on the positive and negative phrased groups. This resulted in all positive factor loadings making it easier to understand their correlation to vaccine uptake.

The analysis was accomplished with R version 4.1.0 (18 May 2021). Microsoft Excel was also used to organize survey data and perform simple calculations.

### 2.3. Stage Three: Data Collection and Analysis of Open-Ended Questions

In the administered survey (Questionnaire S1—Supplementary Materials), managers were asked to comment on factors that, in their experience, increased or decreased uptake of the vaccine by residents and staff and the impact of social media on the vaccination programme. The following questions were asked: (1) Were there any other factors that increased the uptake of the vaccine by residents in your care home? (2) Were there any other factors that decreased the uptake of vaccine by residents in your care home? (3) Were there any other factors that increased the uptake of vaccine by staff? (4) Were there any other factors that decreased the uptake of vaccine by staff? And (5) Are there any approaches you feel may be helpful moving forward in relation to using and managing social media?

We received 48 descriptive responses to open-ended questions from respondents, illustrating positive and negative factors that affected the uptake of the vaccine by staff and residents. Moreover, respondents highlighted the approaches that can be used in managing social media messages. Codes relating to this data were identified and presented in Tables S3–S7 of the Supplementary Materials.

This analysis section is divided into three parts: (i) the uptake of vaccine by residents, (ii) the uptake of vaccine by staff, and (iii) managing social media in promoting the vaccine.

## 3. Results

### 3.1. Quantitative Questionnaire Data 

The study included 67 care home respondents, of which 56.7% and 43.3% were nursing and residential, respectively. The participating care homes were distributed across the five health and social care boards in NI, and the majority (94%) belonged to the independent sector. Additional information about the type of care provided and care home size is provided in Table 2.

Results regarding COVID-19 vaccination uptake by residents showed that residents believed that the vaccine would help life to return to normal (µ = 4.95, *p* < 0.001), relatives of residents were keen for their family to be given the vaccine (µ = 5.24, *p* < 0.0001), residents were keen to take the vaccine (µ = 4.98, *p* < 0.001), residents and their relatives were kept informed by the care home (µ = 5.41, *p* < 0.0001), and residents and their families trusted the opinion of the care home manager regarding the vaccination programme (µ = 4.75, *p* < 0.0001; Table 3). Net Agreement Scores are also presented in Table 3.

In relation to uptake level of COVID-19 vaccination programme by staff, respondents agreed with the following statements: it is important that management encourage and support staff to take the vaccine (µ = 4.94, *p* < 0.0001), staff concerns regarding side effects of the vaccine were a barrier to uptake (µ = 4.56, *p* < 0.0001), and staff under 40 years were less likely to take the vaccine (µ = 4.15, *p* = 0.014). However, participants disagreed with the following: Vaccination date on a non-working day for staff was a barrier for vaccine uptake (µ = 2.58, *p* = 0.003), staff felt it was not safe to go for vaccination because social distancing requirements were not fully met (µ= 2.55, *p* = 0.001), vaccinating staff around their working hours was difficult (µ =2.78, *p* = 0.048), and female staff were more likely to take the vaccine than males (µ = 2.59, *p* = 0.008; Table 4). Net agreement scores are also presented in Table 4.

Analysis of the impact of social media and source of information on uptake level of the COVID-19 vaccination programme by staff and residents showed an agreement with the following statements: social media information about vaccines negatively affected the vaccination programme for staff (µ =4.32, *p* = 0.001), social media is an efficient method of communication by health organisations to staff (µ = 3.95, *p* = 0.043), and social media information about the safety of the vaccine negatively affected vaccination programme for staff (µ = 4.29, *p*= 0.001). However, participants disagreed with the following: social media information about vaccines negatively affected vaccination programme for residents (µ = 2.64, *p* = 0.011), different brands of vaccines made the decision to get vaccinated difficult for residents (µ = 2.58, *p* = 0.006), and social media information about the safety of the vaccine negatively affected the vaccination programme for residents (µ = 2.60, *p* = 0.001; Table 5). Net agreement scores are also presented in Table 5.

Results for the implementation of the COVID-19 vaccination programme showed agreement with several statements as shown in Table 6. However, participants expressed disagreement with the following statements: there was a delay in the 2nd round of vaccinations (µ = 2.59, *p* = 0.005), and the available facilities were not appropriate for the programme (µ = 2.59, *p* = 0.003). Net agreement scores are also presented in Table 6.

The overall evaluation of the COVID-19 vaccination programme in participating care homes was positive, and this was statistically significant when tested against the hypothesis of µ > 3.99 (µ = 4.24, 95% UCL/LCL =3.85 to 4.60, *p* < 0.0001, Net Agreement Score= 73.13). The Likert scale for overall evaluation of the COVID-19 programme is 1 = Strongly negative to 5 = Strongly positive.

Results for testing correlations between staff and residents’ motivation, social media, safety, and programme and process factor constructs are shown in Table 7. The overall assessment of social media questions (separated into questions with positive and negative connotations) and safety questions were shown to be associated with staff motivation for vaccine uptake, i.e., respondents strongly agreed that social media was an effective method of communication but also effective at spreading negative messaging. No associations were observed with social media, safety, and residents’ uptake. The implementation processes for the vaccination programme were associated with vaccine uptake for both staff and residents (Table 7).

**Figure 1:**
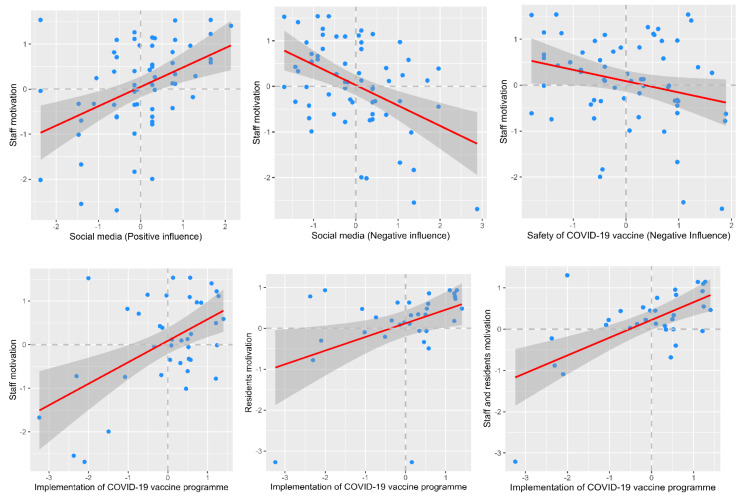
Plots for the relationship between staff and residents’ motivation, social media, safety, and programme and process.

Graphs representing the determined correlations between staff and residents’ motivation, social media, safety, and programme and process are shown in Figure 1.

### 3.2. Open Ended Questions

#### 3.2.1. The Uptake of Vaccine by Residents 

According to care home managers, residents were motivated to receive the vaccine because they wanted to *return back to normal* and allow visits from families. For example, respondent 4 said that the vaccine is *“the hope of being physically closer to a loved one”.* This agreed with respondent 42, who mentioned that vaccine creates the *“desire to be reunited with their families through visitation and day trips”.* Allowing visits was also suggested by respondents 53 and 59. For example, respondent 59 mentioned that *“families* [are] *hopeful that visiting would resume to a normal level”*. From the quotations in Table S3 (Supplementary Materials), the definition of going back to normal by residents is “emotional” by being more attached to family and allowing visits. Based on these quotations, Theme One is “the motivation to uptake the vaccine by residents is to enable family visits”.

Some care home managers highlighted the importance of communication and the relationship between residents/relatives and staff to meet their expectations. For example, respondent 14 said *“good communication between residents, relatives and staff”*. This was in agreement with respondent 30 who explained that *“communication, visually seeing others take the vaccine helped”*. Respondent 40 mentioned *“strong support, relationship and trust between residents and care home manager/staff”* can help in uptake of the vaccine. Based on these quotations, Theme Two is “residents are positively influenced by the relationship with managers and staff to uptake the vaccine”. This theme is justified by respondents 52 and 54 who highlighted the importance of trust in staff because they are also taking it. For example, respondent 52 mentioned *“The fact that staff were also getting it was a reassurance to residents and their families”*. Respondent 55 mentioned another description of trust that staff are available if needed and they said they were *“reassured that staff are on duty 24 h per day if they were feeling unwell”*.

Some quotations also identify factors that decrease the uptake of the vaccine by residents due to the side effects of the vaccine and the uncontrollable delays in the process (Table S4).

Concern regarding the side effects of the vaccine was one of the negative factors that care home managers highlighted. Their *“fear* [of] *the side effects”* was mentioned by respondent 38, and respondent 66 mentioned that some residents experienced *“previous allergies”* with vaccines prior to the COVID-19 pandemic. Care home managers justified this by trust in the vaccine as a concept. For example, respondent 23 said that some residents *“never availed of any vaccine”* and respondent 48 mentioned residents who did not take the vaccine are “*only those residents who would have refused the Influenza Vaccine in the past”*. Based on the quotations, Theme Three is “fear of the unknown hinders the uptake of the vaccine by residents”.

From another point of view, uncontrollable delays in the process were mentioned, such as delays by the GPs and admission/discharge processes. The delay by the GPs was raised by respondent 29, who said there was a *“delay by GPs to come to the home to vaccinate residents”*. Moreover, resident 35 explained that there is a *“lack of GP support”*. This was agreed with by respondent 42 who said, “*uncooperative GP in the case of one resident who has no capacity to give consent*”. This was supported by respondent 52 who mentioned that *“mainly the paperwork for consents, which is always off putting for any activity”*. Based on these quotations, Theme Four is “the uptake of the vaccine by residents is influenced negatively by inefficient practices and processes”.

**Figure 2:**
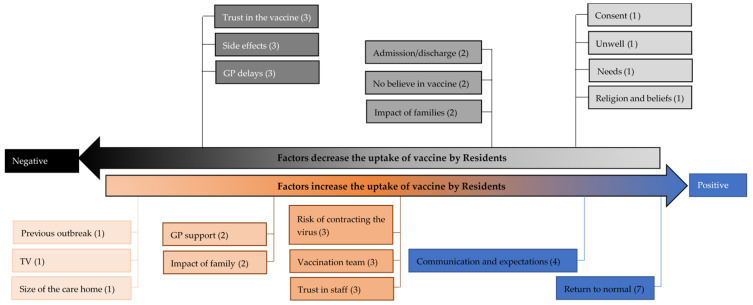
Positive and negative codes that affect the uptake of vaccine by residents.

The positive and negative codes that affected the uptake of vaccine by residents are summarised in Figure 2. The highest number of positive codes related to “return back to normal”, and highest number of negative codes related to “trust in the vaccine”, “safety” and “GP delays”.

#### 3.2.2. The Uptake of Vaccine by Staff

The managers’ views regarding the uptake of the vaccine by staff differ from those of residents (Tables S5 and S6—Supplementary Materials). Despite “back to normal” being the first code, this related to job demands and management encouragement. Respondent 23 mentioned that staff would “*return to normal life”*. Respondent 17 mentioned the same comment *“hope of ending lockdown and restrictions”*. A few managers mentioned that staff are very keen to travel, and staff would like to take holidays and they explained why staff would take the vaccine. For example, respondent 66 said “*they were concerned they wouldn’t be able to go on holiday*”. Respondent 25 mentioned *“the idea of needing a vaccine to go on holiday, pub etc”*. Returning back to normality was explained by respondent 34 who mentioned *“staff had a moral and professional responsibility to receive the vaccine”*, whereas respondent 11 said *“they [staff] felt they had to or it would be frowned upon, the possibility of losing their jobs”*. Moreover, respondent 42 mentioned that *“a relaxation of the weekly Covid testing and wearing of PPE at all times if you were vaccinated which turned out to be untrue”*. A few respondents referred to the impact of previous outbreaks (respondents 4, 5 and 22; Table S5). For example, respondent 4 said *“the potential of protection for our residents and themselves, as a team we managed a challenging outbreak and never ever want to go through such an awful experience again”*. Based on the above quotations, Theme Five is “vaccine is the staff’s ticket to return to normal life at work”.

Encouragement was mentioned as a motivational factor to uptake the vaccine. For example, respondent 15 highlighted the impact of encouragement and said, *“encouragement by Management is seen as for the greater good”*. Respondent 7 mentioned the importance of *“trust in management”*. Respondent 54 mentioned that encouragement can be achieved by seeing *“the manager taking it* [the vaccine]*”*. Respondent 52 mentioned some actions that were taken to encourage staff to uptake the vaccine, and said *“all getting it together here in the Home on the same day—we created a bit of an atmosphere and offered staff breakfast while they were waiting before/after”.* Furthermore, respondent 50 mentioned *“Home Manager rang each staff member individually and encouraged staff to have the vaccine and answered any queries”*. Based on the above quotations, Theme Six is “vaccine uptake by staff is promoted through encouragement and role modelling of care home managers”.

Managers explained that staff are concerned about the safety of the vaccine (Table S6—Supplementary Materials). Safety was described by different care home managers in terms of side effects, blood clots, effectiveness, pregnancy, and fertility. For example, respondent 53 mentioned that *“young staff heard about fertility issues staff pregnant and with allergies”*. This was supported by respondent 6 and 17. Respondent 11 summarised issues associated with the vaccine and said *“younger staff followed the PHA advice at the time regarding pregnancy or planning pregnancy, the speed of the roll out, previously having Covid and having anti-bodies, the emergency approval element, no long-term data, censorship of other professionals with their findings, hypothesis, inadequate debunking of other professional opinion, one vaccine being linked to blood clots and then ceased for certain age groups, the data on the yellow card reporting system, other visiting professionals to the home advising they were not taking it, a comment by a GP following the sudden death of a resident, no coercion in the care home”*. Based on these quotations, Theme Seven is “Staff adopted an analytical approach to explore the potential side effects, related to pregnancy and fertility, and efficiency of the COVID−19 vaccine”.

**Figure 3:**
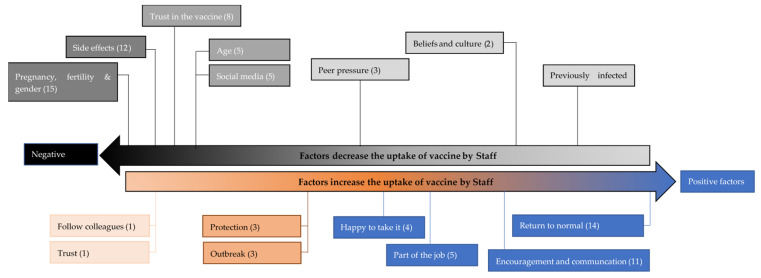
Positive and negative codes that affect the uptake of vaccine by staff.

The positive and negative codes that affected the uptake of the vaccine by staff are summarised in Figure 3. The highest number of positive codes was for return back to normal, and the highest number of negative codes were trust in the vaccine, side effects and its impact on pregnancy, fertility, and gender.

#### 3.2.3. Social Media—Between Managing and Promotion 

Regarding social media, the majority of respondents highlighted the impact of information on the uptake of the vaccine (Table S7—Supplementary Materials). For example, respondent 5 mentioned that “*Social media is beneficial but there is so much in the way of misinformation which leads to negative impact particularly in relation to the pandemic/ vaccination programme”*. Whereas respondent 40 said “*more factual information. Discredit the conspiracy facts with strong scientific evidence, focus on disputing all the concerns as a main point*.” Respondent 47 acknowledged that the use of social media can be considered as a vehicle to deliver information about the vaccine. They illustrated *“clear constant messaging about reading information from trusted sources*”. However, respondent 50 mentioned there is a *“difficulty in ensuring that information on social media is accurate and beneficial for staff”.* Some respondents provided positive comments about the social media, for example, respondent 10 stated that *“social media should have showed more of the positive outcome of the vaccine”*, and respondent 42 expressed that *“medical Consultants, Doctors, Nurses and Scientists should/could have supported the vaccination campaign more actively on Social media”*, but limitations were highlighted by some respondents. For example, respondent 58 said *“the social media message will probably get drowned out by the plethora of misinformation”*. Respondent 4 recommended stricter law enforcement should be applied on social media and mentioned *“Better law to remove false information and a quicker approach to dispelling myths”*. Theme Eight is “Promotion of vaccine uptake through social media requires a consistent evidence-based approach by health authorities to positively influence staff”. Identified themes are presented in Table 8.

The messages on social media can be divided into two types: encouraging or discouraging messages on vaccine uptake. The credibility of information provided has a role in the extent to which people perceive (or believe) these messages. Managers proposed that laws should be enforced to prevent discouraging messages from being promoted.

**Figure 4:**
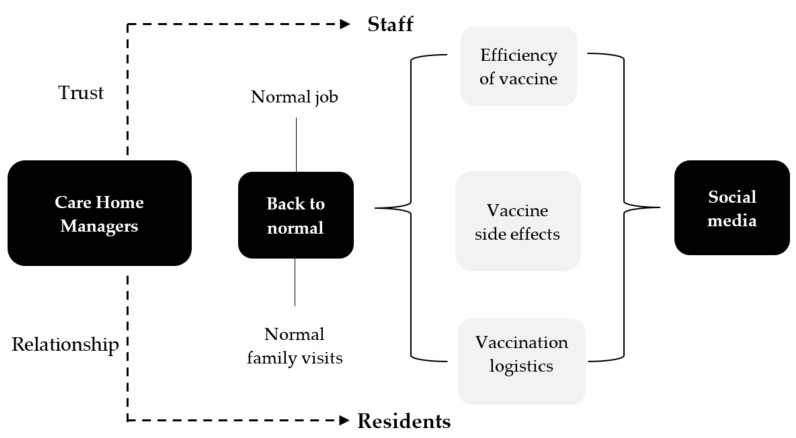
Care Home manager challenges in improving COVID-19 vaccine uptake.

Figure 4 summarises the balance between returning back to normal for staff and residents and the impact of social media on the efficiency of the vaccine, side effects and the vaccination process. The results show the managers’ awareness of all these challenges that they face at care homes in Northern Ireland. This indicates an intelligent leadership approach in handling staff and residents and addressing their concerns.

## 4. Discussion 

Vaccinations are one of the greatest public health successes in history which has contributed to disease prevention and saving lives [30]. While the rapid development of vaccines against COVID-19 is a remarkable achievement, ensuring that enough individuals are vaccinated is crucial to attaining herd immunity requiring vaccination of a very substantial proportion of population, therefore posing a major challenge [30,31]. People’s acceptance of vaccination is determined by many factors as identified earlier by the WHO Strategic Advisory Group of Experts (SAGE) on vaccination (2014) including: (a) confidence or trust in the vaccines and/or provider, (b) complacency (people do not perceive a need for a vaccine) and (c) convenience (access to vaccine) [31,32,33,34]. Of note, hesitancy about COVID-19 vaccination has become evident at a global level [35].

The aim of this study was to explore the experience of care home managers on the implementation and uptake of the COVID-19 vaccination programme by residents and staff in care homes in Northern Ireland.

Quantitative analysis showed that residents and their relatives were keen to take the vaccine. Residents believed that the vaccine would help life to return to normal, and residents and their families trusted the care home manager’s opinion regarding the vaccination programme. The analysis of open-ended questions for residents’ vaccine uptake identified four themes. These include residents’ emotions reflecting a desire to go back to normal, maintain family visits and reduce restrictions (Theme One), and of being influenced by their relationship with managers and staff to take the vaccine (Theme Two). Based on the number of determined codes, these themes may have a stronger impact on residents’ vaccine uptake. Previous research explains the link between family involvement and residents as a form of informal care and how family caregiving can improve the care outcomes [36]. Themes Three and Four were related to uncontrollable factors (e.g., vaccine side effects, delays in the process) that managers can consider how to mitigate in future. Few respondents mentioned the religious views and beliefs of the residents as barriers to uptake of the vaccine and this may warrant further future assessment. Another area of future study would be consideration of the impact of previous vaccination programmes (for example Flu) upon the uptake of the Covid-19 vaccination.

In relation to staff, analysis of quantitative findings showed that management encouragement and support was important to vaccine uptake. Staff concerns regarding side effects of the vaccine were a barrier to uptake, and staff under 40 years were noted as being less likely to take the vaccine. In a cross sectional study, involving a sample from the UK adult population, concerns about future unforeseen side effects were one of the most important determinants of both uncertainty and unwillingness to vaccinate against COVID-19 [22]. The analysis of open-ended questions for staff vaccine uptake in this study identified another three themes. The managers highlighted the impact of returning back to normal by staff (Theme Five). Care home work can be stressful and difficult due to long working hours and high duty of care expectations from management, the families of the residents, and the health care authority [37]. This may affect the physical and mental wellbeing of staff which in turn may also affect the quality of service and care provided [37]. In addition, some care home managers referred to the impact of previous outbreaks on staff motivation towards taking the vaccine. The desire not to go through the stressful situation created by outbreaks, was one of the factors that increased vaccine uptake. The impact of outbreaks on care homes and the importance of protection for care homes against SARS-CoV-2 infection has been demonstrated by other studies [38]. In this research, care home managers mentioned that staff would like to return back to their normal jobs and be able to book holidays as normal to minimize anxiety because of COVID-19 as mentioned in Theme Five. Theme Six highlighted the importance of trust in management in encouraging vaccine uptake by staff. Finally, staff acceptance of vaccination was found to be dependent mainly on scientific evidence (theme seven). For example, staff are more concerned about the safety and efficiency of the vaccine as mentioned in Theme Seven.

The overall evaluation of the COVID-19 vaccination programme in the participating care homes was positive. Care home managers agreed that the vaccination programme and process was well organized, safety measures were implemented in the programme, and maintaining the practice of routine testing for COVID-19 was important. They also agreed that the information provided to residents, relatives, and staff about COVID-19 vaccination was appropriate. According to care home managers, the vaccination programme and logistical processes were found to be positively related to the motivation to uptake the vaccine. This is in line with other recommendations to promote COVID-19 vaccine uptake, which emphasized a professional approach in planning and delivering the COVID-19 vaccination programme [39].

The quantitative data tested the relationship between the motivation of staff and residents for vaccines uptake with social media, and safety (side effects). No associations were observed with social media and safety concerns in terms of residents’ COVID-19 vaccine uptake. This may be explained by the limited access of residents to social media platforms within the care homes. For staff, it was found that vaccine uptake is negatively influenced by the perceived side effect. This is consistent with other findings reported elsewhere [22,31,40]. The common reported side effects were published by the Medicines and Healthcare products Regulatory Agency (MHRA) who highlighted that reports of serious side effects remain very rare [41].

The perceived side effects of the vaccine may also be influenced by social media and misinformation. The quantitative results of the present study indicated that staff can be motivated by social media, but it can also be a demotivating factor. In addition, analysis of open-ended questions recognized the important role of social media in disseminating the correct information about the vaccine (Theme Eight). Social media has become increasingly used as a source for searching health information [42,43]. However, the implementation of the vaccination programme faced significant challenges with the rise of misinformation that fills knowledge voids under conditions of uncertainty [44,45,46]. Misinformation can impact confidence in the vaccine, despite the accessibility of the vaccine to both residents and staff [47,48,49]. The role of coherent media presence in supporting the vaccination programme, via delivering consistent messaging and challenging misinformation, has also been emphasized in other studies [31,35,43,45,50,51].

This study is the first to explore care home managers experience on the implementation and uptake of the COVID-19 vaccines in care homes. The study has the strength of using an exploratory mixed methods approach, i.e., semi-structured interviews to inform and derive the administered survey. This was supported by the analysis of open-ended questions that were integrated within the administered survey. However, the study has some limitations. The impact of residents’ culture and social interaction between staff and residents on the uptake of the vaccine was not assessed in this study and may benefit from further future work. In addition, the survey may have benefited from a larger sample size if it was possible. It is recognised this study was undertaken during a time of unprecedented demand upon the care home sector impacting upon the capacity of the care home managers. Nevertheless, we estimated the maximum margin of error (MOE) for this survey to be 9.35%. With this attained MOE, we are 90% confident that the consensus opinions are true and not a result of random chance. Furthermore, in September 2021, the UK announced that the most vulnerable people will be offered COVID-19 booster vaccines, which can be another area for future research in care homes. The study did not evaluate the concerns for the health system’s capacity to accommodate and treat larger than usual amounts of patients on vaccination uptake. It is important to note that COVID-19 and poor vaccine uptake are jeopardizing the viability of many health care systems having to cope with limited ventilators, overwhelmed ICUs, and an influx of patients seeking treatment for COVID-19 infection [52,53].

## 5. Conclusions

This study showed that residents are emotionally influenced to be vaccinated so they can return to normal as defined by their ability to meet their families, whereas staff are motivated by job demands and their responsibilities to minimise the risk to residents and their families. It is important these factors are integrated into further vaccine campaigns to support positive engagement and uptake with vaccine programmes. The relationship between residents, staff, and managers was found to be critical to motivate both staff and residents to be vaccinated. Staff were found to be more concerned about the scientific aspects of the vaccine when compared with residents. Social media was found to be an important vehicle to control and provide credible information about the vaccine and safety. Social media can be used positively to reduce the uncertainty and provide accurate information about the vaccine uptake. This study highlights the importance of managers’ leadership style in handling the challenges through trust and relationship with staff and residents. The findings identified challenges to the uptake of the COVID-19 vaccine by staff and residents that can inform the implementation of future vaccination programmes.

## Figures and Tables

**Table 1:** Estimated Cronbach’s alpha tests for the administered questionnaire.

Focus Sections	Items	Cronbach’s Alpha
Section B—Vaccine uptake by residents	9	0.700
Section C—Vaccine uptake by staff	15	0.677
Section D—Social media	10	0.741
Section E—Vaccination programme	16	0.826

**Table 2:** Study characteristics of participating care homes (*n* = 67).

**Characteristics**	**Number of Care Homes**	**Percentage of Care Homes**
**Facility Type**		
Nursing	38	56.7%
Residential	29	43.3%
**Care Home Location**		
South Eastern HSC Trust	19	28.4%
Belfast HSC Trust	18	26.9%
Northern HSC Trust	13	19.4%
Southern HSC Trust	13	19.4%
Western HSC Trust	4	6.0%
**Care Home Ownership Type**		
Independent sector	63	94.0%
HSC Trust	4	6.0%
**Care Type**		
Dementia/frail elderly	38	56.7%
General nursing	22	32.8%
Learning Disability	15	22.4%
Physically dependent under 65 years	14	20.9%
Elderly Mentally Infirm	6	9.0%
Brain Injury	3	4.5%
Mental Health	2	3.0%
General Residential Care	2	3.0%
Physical Disability	1	1.5%
Intermediate Care	1	1.5%
Traumatic Injury	1	1.5%
**Care Home Size**		
30 persons	29	43.3%
31–50 persons	25	37.3%
More than 51 persons	13	19.4%

**Table 3:** Analyses of uptake level of COVID-19 vaccination programme in care homes by residents.

Question Number	Questions	Mean	LCL (95%)	UCL (95%)	*p*-Value (Mean < 3.0)	*p*-Value (Mean ≥ 4.0)	Net Agreement Score
Q6	Residents believed that the vaccine would help life to return to normal	4.95	4.28	5.50	0.999	<0.0001	78.18
Q7	Vaccination process for dementia residents was a challenge	3.65	3.06	4.28	0.999	0.723	1.96
Q8	Relatives of residents were keen for their family to be given the vaccine	5.24	4.53	5.85	0.999	<0.0001	78.79
Q9	Residents were keen to take the vaccine	4.98	4.33	5.53	0.999	<0.0001	79.31
Q10	Residents and their relatives were kept informed by the Care Home	5.41	4.70	5.89	0.999	<0.0001	84.13
Q11	Signing/organising consent forms was difficult	3.57	3.00	4.17	0.999	0.825	−4.76
Q12	The influence of relatives was an important driver for vaccine uptake by residents	3.98	3.26	4.74	0.999	0.110	23.08
Q13	Residents and their families trusted the opinion of the Care Home manager regarding the vaccination programme.	4.75	4.00	5.40	0.999	<0.0001	61.19
Q14	Relatives of residents had concerns about the safety of vaccine	3.25	2.65	3.84	0.914	0.999	−25.00

**Table 4:** Analyses of uptake level of COVID-19 vaccination programme in care homes by staff.

Question Number	Questions	Mean	LCL (95%)	UCL (95%)	*p*-Value (Mean < 3.0)	*p*-Value (Mean ≥ 4.0)	Net Agreement Score
Q17	It is important that management encourage and support staff to take the vaccine	4.94	4.25	5.55	0.999	<0.0001	64.18
Q18	Vaccination date on a non-working day for staff was a barrier for vaccine uptake	2.58	2.00	3.15	0.003	0.999	−63.64
Q19	Staff believe that the vaccine alone will facilitate activities that care homes were able to engage with pre-COVID	3.77	3.20	4.35	0.999	0.406	15.15
Q20	Filling the forms for vaccination is time-consuming	3.94	3.37	4.50	0.999	0.085	15.15
Q21	Staff felt it was not safe to go for vaccination because social distancing requirements were not fully met	2.55	2.00	3.05	0.001	0.999	−75.76
Q22	Staff required continued motivation by the management to take the vaccine	3.79	3.10	4.50	0.999	0.399	12.12
Q23	Staff concerns regarding side effects of the vaccine were a barrier to uptake	4.56	4.00	5.10	0.999	<0.0001	63.64
Q24	Some staff discouraged other staff from taking the vaccine	3.70	3.10	4.35	0.999	0.571	4.48
Q25	Managers felt that staff were influenced in the decision to take the vaccine by what their peers decided to do	3.97	3.35	4.55	0.999	0.068	27.27
Q26	Signing/organising consent forms was difficult	3.24	2.70	3.80	0.918	0.999	−31.34
Q27	Staff believe there was unclear information regarding vaccine effectiveness	3.68	3.10	4.35	0.999	0.606	0.00
Q28	Staff believe there was unclear information regarding vaccine safety	3.79	3.15	4.40	0.999	0.409	9.09
Q29	Vaccinating staff around their working hours was difficult	2.78	2.30	3.26	0.048	0.999	−68.75
Q30	Female staff were more likely to take the vaccine than males	2.59	2.07	3.14	0.008	0.999	−79.66
Q31	Staff under 40 years were less likely to take the vaccine	4.15	3.45	4.79	0.999	0.014	33.33

**Table 5:** Analyses of the impact of social media and source of information on uptake level of COVID-19 vaccination programme in care homes by staff and residents.

Question Number	Questions	Mean	LCL (95%)	UCL (95%)	*p*-Value (Mean < 3.0)	*p*-Value (Mean ≥ 4.0)	Net Agreement Score
Q34	Social media information about vaccines negatively affected vaccination programme for residents	2.64	2.11	3.21	0.011	0.999	−62.50
Q35	Social media information about vaccines negatively affected vaccination programme for staff	4.32	3.75	4.85	0.999	0.001	48.48
Q36	Social media is an efficient method of communication by health organisations to staff	3.95	3.30	4.56	0.999	0.043	40.63
Q37	Social media is an efficient method of communication by health organisations to residents	3.02	2.47	3.58	0.497	0.999	−42.86
Q38	Different brands of vaccines made the decision to get vaccinated difficult for staff	3.76	3.16	4.35	0.999	0.546	−3.03
Q39	Different brands of vaccines made the decision to get vaccinated difficult for residents	2.58	2.05	3.17	0.006	0.999	−83.05
Q40	Social media information about the safety of the vaccine negatively affected vaccination programme for residents	2.60	2.06	3.22	0.010	0.999	−76.67
Q41	Social media information about the safety of the vaccine negatively affected vaccination programme for staff	4.29	3.68	4.90	0.999	0.001	48.48
Q42	Social Media was a useful platform to support the vaccination campaign	3.71	3.00	4.30	0.999	0.518	26.15
Q43	The support of vaccine by celebrities on social media helped to increase uptake of staff	3.34	2.80	3.89	0.985	0.993	−25.00

**Table 6:** Analyses of the implementation of the COVID-19 vaccination program in care homes.

Question Number	Questions	Mean	LCL (95%)	UCL (95%)	*p*-Value (Mean < 3.0)	*p*-Value (Mean ≥ 4.0)	Net Agreement Score
Q45	The vaccination programme was well organised	4.69	3.95	5.30	0.999	<0.0001	64.18
Q46	There was a delay in the 2nd round of vaccinations	2.59	2.00	3.15	0.005	0.999	−72.73
Q47	The programme was 100% voluntary	4.99	4.30	5.50	0.999	<0.0001	70.15
Q48	Timeframe for vaccinating all staff and residents was a challenge	3.47	2.90	4.10	0.998	0.947	−18.18
Q49	The available facilities were not appropriate for the programme	2.59	2.11	3.11	0.003	0.999	−78.79
Q50	Safety measures were implemented in the programme	4.94	4.25	5.45	0.999	<0.0001	70.15
Q51	The second dose of vaccine was associated with more side effects	4.30	3.60	5.00	0.999	0.002	48.48
Q52	It is important to maintain the practice of routine testing for COVID-19	4.75	4.00	5.35	0.999	<0.0001	64.18
Q53	Technological difficulties (booking online) had a negative impact on the programme	3.47	2.84	4.17	0.996	0.906	−23.33
Q54	Appropriate information was provided on what the Care Home needed to do	4.77	4.15	5.30	0.999	<0.0001	75.76
Q55	The vaccination team was sensitive to the needs of the Care Home	4.98	4.37	5.58	0.999	<0.0001	72.31
Q56	Social distancing adherence was emphasised during the vaccination day(s)	4.85	4.10	5.50	0.999	<0.0001	66.67
Q57	The information provided to residents about COVID-19 vaccination was appropriate	4.67	3.89	5.32	0.999	<0.0001	58.73
Q58	The information provided to staff about COVID-19 vaccination was appropriate	4.65	3.95	5.25	0.999	<0.0001	60.61
Q59	The information provided to relatives about COVID-19 vaccination was appropriate	4.72	4.10	5.35	0.999	<0.0001	68.75
Q60	The posters/signage provided for the vaccination day(s) were appropriate	4.57	3.82	5.20	0.999	<0.0001	65.52

**Table 7:** Assessment of correlations between staff and residents’ motivation, social media, perceived safety, and programme and process.

**Staff motivation construct**		
**Overall Drivers of Uptake for Staff**	**Correlation**	***p*-value**
Social Media (Positive influence) *	0.431	0.001
Social Media (Negative influence) **	−0.454	<0.0001
Programme and process	0.526	0.001
Perceived safety (Negative influence)	−0.254	0.045
**Resident motivation construct**		
**Overall Drivers of Uptake for Residents**	**Correlation**	***p*-value**
Programme and process	0.410	0.018
**Overall motivation for staff and residents construct**		
**Overall Drivers of Uptake for Staff and Residents**	**Correlation**	***p*-value**
Programme and process	0.619	<0.0001

* Q36 and Q43; ** Q35, Q38, and Q41.

**Table 8:** Summary of identified themes regarding the uptake of the vaccine by residents and staff, and the impact of social media.

**Managers’ view regarding the uptake of the vaccine by Residents**	
Positive Themes	Negative Themes
The motivation to uptake the vaccine by residents is family visits Residents are positively influenced by relationship with managers and staff to uptake the vaccine	Fear of unknown hinders the uptake of the vaccine by residents The uptake of the vaccine by residents is influenced negatively by inefficient practices and processes
**Managers’ view regarding the uptake of the vaccine by Staff**	
Positive Themes	Negative Themes
Vaccine is the staff’s ticket to return to normal life at work Vaccine uptake by staff is promoted through encouragement and role modelling of care home managers	Staff adopted an analytical approach to explore the potential side effects, related to pregnancy and fertility, and efficiency of the COVID-19 vaccine
**The Impact of Social Media**	
Promotion of vaccine uptake through social media requires a consistent evidence-based approach by health authorities to positively influence staff	

## Data Availability

All collected data for this study was published in this article.

## Supplementary Materials

The following are available online at https://www.mdpi.com/article/10.3390/vaccines9101160/s1: Questionnaire S1 Survey of the experience of care homes managers of COVID-19 vaccination programme implementation and uptake by residents and staff; Semi-structured guide S1 A semi-structured guidance for each interview session; Consent form S1 Participants information sheet and consent form; Invitation letter S1 the invitation letter which was emailed to all care homes managers in Northern Ireland; Table S1 Summary of codes from semi-structured interviews; Table S2 Questions included in testing H1, H2, and H3 hypotheses; Table S3: Codes and frequencies for other factors that increased the uptake of vaccine by residents in care homes (Q15 in the questionnaire); Table S4 Codes and frequencies for other factors that decreased the uptake of vaccine by residents care homes (Q16 in the questionnaire); Table S5 Codes and frequencies for other factors that increased the uptake of vaccine by staff (Q32 in the questionnaire); Table S6 Codes and frequencies for other factors that decreased the uptake of vaccine by staff (Q33 in the questionnaire); Table S7 Codes and frequencies for approaches that would be helpful moving forward in relation to using and managing social media (Q44 in the questionnaire); and Figure S1: An illustration of the codes from seven semi structured interviews.
