# Supplementary material for: Exploration of the Experience of Care Home Managers of COVID-19 Vaccination Programme Implementation and Uptake by Residents and Staff in Care Homes in Northern Ireland
Source: Vaccines (Basel). 2021 Oct 10;9(10):1160. doi: 10.3390/vaccines9101160 (PMC8539315; doi:10.3390/vaccines9101160)

Questionnaire S1 Survey of the experience of care homes managers of covid-19 vaccination programme implementation and uptake by residents and staff

We are conducting a survey that aims to explore the experience of Care Home Managers of the COVID-19 Vaccination Programme. The information gained will be used to inform recommendations for the implementation of the vaccine programme moving forward and help address any issues that may have arisen during its implementation to date. It is hoped that the lessons learned will support ongoing service quality and safety improvements for older people in Care Homes. In addition, the project may also help in providing recommendations that can be used by Care Homes and other organisations in delivering future COVID-19 vaccine programmes, informing use of available resources.

All information which is collected will be anonymous and no personal data or identifiers will be recorded or stored. It will not be possible to link any particular answer to any participant or Care Home. All collected anonymous data will be stored in password-protected computers and only the study team will have access to this data. The study was developed and reviewed by the Public Health Agency in Northern Ireland and the University of Huddersfield in England. Results of the study will be shared with Care Home Providers in NI, disseminated within relevant groups who are in charge of COVID-19 Vaccination Programme, and within peer-reviewed scientific journals and conferences.

Please answer the following questions to the best of your ability and as fully as possible.

The survey should take approximately 15-20 minutes to complete. Please complete the questionnaire by Monday 21st June.

If you need assistance accessing or completing this survey you can contact the Public Health Agency on 028 9536 2869.

By completing this survey we are assuming that you are giving your consent to participation on this study.

THANK YOU for your time and for contributing to this valuable piece of work

Section A: About your Care Home

This section refers to general information about your Care Home. Please answer all questions

1. How would you describe the type of facility you work in? (please select one)
   - ☐ Nursing
   - ☐ Residential

2. Where in Northern Ireland is your Care Home located? (please select one)
   - ☐ Belfast HSC Trust
   - ☐ Northern HSC Trust
   - ☐ South Eastern HSC Trust
   - ☐ Southern HSC Trust
   - ☐ Western HSC Trust

3. How would you describe the ownership of your Care Home? (please select one)
   - ☐ HSC Trust
   - ☐ Independent sector

4. How would you best describe the care provided in your facility (please select all that apply)
   - ☐ General nursing
   - ☐ Physically dependent under 65 years
   - ☐ Dementia/frail elderly
   - ☐ Elderly Mentally Infirm (EMI)
   - ☐ Other – please specify ……………………………………………………………………………………………

5. What is the size of your Care Home? (please select one)

   - ☐ 30 persons
   - ☐ 31-50 persons
   - ☐ More than 51 persons

Section B: About vaccine uptake by residents

This section refers to the uptake level of COVID-19 vaccination programme in Care Home by RESIDENTS.

To what extent do you agree with the following statements?

	Item	Strongly disagree	Disagree	Somewhat disagree	Somewhat agree	Agree	Strongly agree	N/A
6	Residents believed that the vaccine will help life to return to normal							
7	Vaccination process for dementia residents was a challenge							
8	Relatives of residents were keen for their family to be given the vaccine							
9	Residents were keen to take the vaccine							
10	Residents and their relatives were kept informed by the Care Home							
11	Signing/organising consent forms was difficult							
12	The influence of relatives was an important driver for vaccine uptake by residents							
13	Residents and their families trusted the opinion of the Care Home manager regarding the vaccination programme.							
14	Relatives of residents had concerns about the safety of vaccine							

15. Were there any other factors that **increased** the uptake of vaccine by residents in your Care Home?

……………………………………………………………………………..

………………………………………………………………………………

………………………………………………………………………………..

……………………………………………………………………………..

16. Were there any other factors that **decreased** the uptake of vaccine by residents in your Care Home?

……………………………………………………………………………..

………………………………………………………………………………

……………………………………………………………………………..

……………………………………………………………………………..

Section C: About vaccine uptake by staff

This section refers to the uptake level of COVID-19 vaccination programme in Care Home by STAFF.

To what extent do you agree with the following statements?

	Item	Strongly disagree	Disagree	Somewhat disagree	Somewhat agree	Agree	Strongly agree	N/A
17	It is important that management encourage and support staff to take the vaccine							
18	Vaccination date on a non-working day for staff was a barrier for vaccine uptake							
19	Staff believe that the vaccine alone will facilitate activities that care homes were able to engage with pre COVID							
20	Filling the forms for vaccination is time-consuming							
21	Staff felt it was not safe to go for vaccination because social distancing requirements were not fully met							
22	Staff required continued motivation by the management to take the vaccine							
23	Staff concerns regarding side effects of the vaccine were a barrier to uptake							
24	Some staff discouraged other staff from taking the vaccine							
25	Managers felt that staff were influenced in the decision to take the vaccine by what their peers decided to do							
26	Signing/organising consent forms was difficult							
27	Staff believe there was unclear information regarding vaccine effectiveness							
28	Staff believe there was unclear information regarding vaccine safety							
29	Vaccinating staff around their working hours was difficult							
30	Female staff were more likely to take the vaccine than males							
31	Staff under 40 years were less likely to take the vaccine							

32. Were there any other factors that **increased** the uptake of vaccine by staff?

…………………………………………………………………………..

……………………………………………………………………………

…………………………………………………………………………...

…………………………………………………………………………..

33. Were there any other factors that **decreased** the uptake of vaccine by staff?

…………………………………………………………………………..

……………………………………………………………………………

…………………………………………………………………………..

…………………………………………………………………………..

Section D: About social media

This section refers to the impact of social media and source of information on uptake level of COVID-19 vaccination programme in Care Home by STAFF and RESIDENTS

To what extent do you agree with the following statements?

	Item	Strongly disagree	Disagree	Somewhat disagree	Somewhat agree	Agree	Strongly agree	N/A
34	Social media information about vaccines negatively affected vaccination programme for residents							
35	Social media information about vaccines negatively affected vaccination programme for staff							
36	Social media is an efficient method of communication by health organisations to staff							
37	Social media is an efficient method of communication by health organisations to residents							
38	Different brands of vaccines made the decision to get vaccinated difficult for staff							
39	Different brands of vaccines made the decision to get vaccinated difficult for residents							
40	Social media information about the safety of the vaccine negatively affected vaccination programme for residents							
41	Social media information about the safety of the vaccine negatively affected vaccination programme for staff							
42	Social Media was a useful platform to support the vaccination campaign							
43	The support of vaccine by celebrities on social media helped to increase uptake of staff							

44. Are there any approaches you feel may be helpful moving forward in relation to using and managing social media?

…………………………………………………………………………..

……………………………………………………………………………

…………………………………………………………………………..

…………………………………………………………………………..

Section E: About vaccination programme

This section refers to the implementation of the COVID-19 vaccination programme at the Care Home.
To what extent do you agree with the following statements?

	Item	Strongly disagree	Disagree	Somewhat disagree	Somewhat agree	Agree	Strongly agree	N/A
45	The vaccination programme was well organised							
46	There was a delay in the 2nd round of vaccinations							
47	The programme was 100% voluntary							
48	Timeframe for vaccinating all staff and residents was a challenge							
49	The available facilities were not appropriate for the programme							
50	Safety measures were implemented in the programme							
51	The second dose of vaccine was associated with more side effects							
52	It is important to maintain the practice of routine testing for COVID-19							
53	Technological difficulties (booking online) had a negative impact on the programme							
54	Appropriate information was provided on what the Care Home needed to do							
55	The vaccination team was sensitive to the needs of the Care Home							
56	Social distancing adherence was emphasised during the vaccination day(s)							
57	The information was provided to residents about COVID-19 vaccination was appropriate							
58	The information was provided to Staff about COVID-19 vaccination was appropriate							
59	The information was provided to relatives about COVID-19 vaccination was appropriate							
60	The posters/signage provided for the vaccination day(s) were appropriate							

61. What is your overall evaluation of the COVID-19 vaccination programme?

Strongly positive   Positive   Neutral   Negative   Strongly negative

Thank you for completing this questionnaire

Semi-structured guide S1 A semi-structured guidance for each interview session

Start of interview

• Introduce yourself and thank for participation

• Explain reason for interview:

Successful implementation of a vaccine programme is dependent on adequate levels of uptake. Understanding the views and barriers related to vaccination is important to understand more fully the characteristics associated with lower levels of uptake. This project aims to explore the experience of Care Home Managers of the COVID-19 Vaccination Programme. The information gained will be used to inform recommendations for the implementation of the vaccine programme moving forward and help address any issues that may have arisen during its implementation to date.

• Explain data confidentiality- as per participant information sheet

• Confirm consent to record- as per the consent form

• Agree time limits (approx. 25 minutes)

We may need few questions to collect relevant information about the interviewee, e.g. level of experience

General Experiences to the Vaccination programme	-What were your hopes when your Care Home engaged with the vaccination programme at the first instance?<br><br>- What were your fears when your Care Home engaged with the vaccination programme at the first instance?<br><br>- What challenges did staff face regarding the vaccination programme during implementation?<br><br>- What challenges did residents face regarding the vaccination programme during implementation?

	- What challenges did relatives face regarding the vaccination programme during implementation?<br><br>- From your perspective, what were the positive aspects about the vaccination programme?<br><br>- From your perspective, what were the negative aspects about the vaccination programme?<br><br>- How did the social media impact the vaccination programme in your Care Home?<br><br>- What do you think of the vaccination programme? and why? Please comment on the speed of vaccine delivery and time to be prepared.
Responding to the Outbreak	- Did you realise that you could still get an outbreak even after the vaccination programme? and why?<br><br>-What were the emotional responses of staff when your Care Home experienced an outbreak following the vaccination programme?<br><br>- Have you witnessed emotional responses from residents and their relatives regarding the outbreak following the vaccination programme? What was your experience?<br><br>- If you had an outbreak prior to the vaccination programme was the experience different in any way to that post the vaccination programme? What do you think the reasons were for any differences?<br><br>- What were your interventions or actions in managing the outbreak?

	- What information would have supported you to communicate with staff about the outbreak?<br><br>- What information would have supported you to communicate with residents about the outbreak?<br><br>- What information would have supported you to communicate with relatives about the outbreak?

Closing:

- Are there any other factors that we have not covered, and you think might be important?
- Anything else you would like to add?
- Any questions?

Thanks for your time

Consent form S1 Participants information sheet and consent form

Study Title: Exploration of the impact of COVID-19 Vaccination Programme in Care Homes in Northern Ireland– a study of the lived experience

You are being invited to take part in this study. Before you decide it is important for you to understand why this study is being done and what it will involve. Please take time to read the following information and discuss it with others if you wish. Ask if there is anything that is not clear or if you would like more information. May I take this opportunity to thank you for taking time to read this.

What is the purpose of the project?

Since the emergence of the COVID-19 pandemic, people living and working in Care Homes were significantly impacted. Care-home residents are more vulnerable to infection with an increased likelihood of risk factors, including age, disability, and pre-existing comorbidities.The Department of Health has rolled out a phased COVID-19 vaccination plan in which residents, and staff in Care Homes facilities are the top priority group for the administration of the COVID-19 vaccine. Vaccines have become the hope for better life after the COVID-19 pandemic. Successful implementation of a vaccine programme is dependent on adequate levels of uptake. Understanding the views and barriers related to vaccination is important to understand more fully the characteristics associated with lower levels of uptake.

This project aims to explore the experience of Care Home Managers of the COVID-19 Vaccination Programme. The information gained will be used to inform recommendations for the implementation of the vaccine programme moving forward and help address any issues that may have arisen during its implementation to date. It is hoped that the lessons learned will support ongoing service quality and safety improvements for older people in Care Homes and also increase the public trust in services provided for this population.

In addition, the project may also help in providing recommendations that can be used by Care Homes and other organisations in delivering future Covid-19 vaccine programmes, informing better use of available resources and logistics. Ultimately, the effective implementation of the COVID-19 Vaccination Programme will lead to saving lives and protecting the NHS. Ongoing work to increase the public's confidence and acceptance for the vaccine is central to this aim.

Why am I doing the project?

This study is being conducted as part of the Public Health Agency role to inform public health policies and strategies to improve healthcare. The outcomes of this study will help to inform current and future COVID-19 Vaccination Programmes policies in Care Homes in Northern Ireland.

Do I have to take part?

Participation on this study is entirely voluntary, so please do not feel obliged to take part but we will of course encourage and welcome your involvement. Participants may withdraw from the study at any stage without giving an explanation to the project team.

What do I have to do if I agree to take part?

You will be invited to take part in an interview, which will be delivered using Zoom platform. This will be recorded for the purposes of transcribing the experiences shared. The recording will be stored as per GDPR and only used for the purpose of transcription. It will not be shared outside of the project team. This should take no more than 20-25 minutes of your time.

What are the advantages of taking part?

Taking part in this project will enable the aim of the study to be achieved, and your participation will contribute to supporting improvements in the implementation of COVID-19 Vaccination Programme in Care Homes in Northern Ireland.

Are there any disadvantages to taking part?

There should be no foreseeable disadvantages to your participation. If you are unhappy or have further questions at any stage in the process, please address your concerns initially to the person who is conducting the interview if this is appropriate. Alternatively, please contact the project coordinator (details provided below).

Will all my details be kept confidential?

All information which is collected will be strictly confidential and anonymised before the data is presented in the meetings or publications, in compliance with the Data Protection Act and ethical research guidelines and principles. Interviews discussions will be recorded and transcribed immediately, and then all recorded material will be destroyed. Discussions will be anonymous and no personal data or identifiers will be recorded

or stored. It will not be possible to link any particular comment to any participant or Care Home. All collected anonymous data will be stored in password-protected computers and only the study team will have access to this data.

What happens when the study ends?

The interviews transcripts will be analysed and used to develop a questionnaire which will then be sent to all Care Homes in Northern Ireland. It is anticipated that the overall results of this study will inform the development of policies and strategies to facilitate improvements in the implementation of COVID-19 Vaccination Programme in Care Homes in Northern Ireland.

What will happen to the results of the study?

Results of the study will be shared with Care Home Providers in NI, disseminated within relevant groups who are in charge of COVID-19 Vaccination Programme, and within peer-reviewed scientific journals and conferences.

Who has reviewed and approved the study, and who can be contacted for further information?

The study was reviewed and approved by the Public Health Agency in Northern Ireland.

Name & Contact Details of person conducting the interviews:

Mrs Linda Craig: linda.craig3@hscni.net

Mrs Dalrene Masson: Dalrene.masson2@hscni.net

Name & Contact Details of Project Coordinator:

Heather Reid: heather.reid@hscni.net

Consent form

Study Title: Exploration of the impact of COVID-19 Vaccination Programme in Care Homes in Northern Ireland– a study of the lived experience

Project Coordinator: Heather Reid, Consultant in Public Health, Public Health Agency

Additional investigator: Linda Craig, Regional Lead for Patient Client Experience, PHA
Dalrene Masson, Patient Client Experience Facilitator, PHA

Please confirm, by marking the boxes, that you agree with the following statements:

1.	I have read and understood the information about the project above, as provided in the Participant Information Sheet.	☐
2.	I have been given the opportunity to ask questions about the project and my participation, and have received answers to any questions raised	☐
3.	I voluntarily agree to participate in this project.	☐
4.	I understand I can withdraw at any time without giving reasons and that I will not be penalised for withdrawing nor will I be questioned on why I have withdrawn.	☐
5.	The procedures regarding confidentiality have been clearly explained to me.	☐
6.	I understand that discussions taking place during the interviews will be recorded and then transcribed by the project coordinator's team. I understand that following transcription the recordings will be deleted.	☐
7.	I understand that no comments made by me during interviews discussions will be able to be linked back to me but may be anonymously quoted in subsequent reports / publications. The use of	☐

	the data in research, publications, sharing and archiving has been explained to me.	
8.	I understand that the data will be accessed by the project coordinator's team responsible for conducting this research	☐

Name of participant, signature and date:

Name of person taking consent, signature and date:

Invitation letter S1 the invitation letter which was emailed to all care homes managers in Northern Ireland

Dear Care Home manager,

We are conducting a survey that aims to explore and to learn from the experience of the COVID-19 Vaccination programme roll out from the perspective of Care Home managers. The information gained will be used to inform recommendations for the implementation of the vaccine programme moving forward and help address any issues that may have arisen during its implementation to date. The questionnaire can be accessed via the following link:

https://hud.eu.qualtrics.com/jfe/form/SV_3jEBbb2fy0DbtHg

Please use the link to access the survey, however if you have any issues, we will be happy to receive it by post to the 10,000 More Voices Team at the PHA or by e-mail to 10000morevoices@hscni.net. If you need assistance you can also contact the Public Health Agency on 028 9536 2869. The survey, which has been developed in close collaboration with Care Home managers, should take no more than 20 minutes to complete.

As you will be aware, the COVID-19 Vaccination programme commenced in early December 2020 with the care home sector leading the way to protect some of the most vulnerable individuals in our society. Whilst there is already good evidence to suggest that the COVID-19 vaccine has been very successful in preventing morbidity and mortality, there is still much to understand about how maximise the programme's effectiveness and deliver benefits for both staff and residents across the sector.

It is well recognised that Care Homes hold a wealth of knowledge which, if harnessed, could drive further improvements to the delivery of vaccination programmes in this sector. Please take this opportunity to have your say and influence how vaccination programmes should best be delivered in Care Homes moving forward.

Responses should be returned no later than Monday 21st June 2021.

Stephen Bergin
Director of Public Health
PHA

Rodney Morton
Director of Nursing
PHA

Brendan Whittle
Director of Social Care
HSCB

Table S1 Summary of codes from semi structured interviews

Codes	Number of respondents	Repetition	Question in the questionnaire
Famous people	1	1	Q43
Force	1	1	Q47
Gender	2	2	Q30
Authorisation process	6	16	Q11, Q20, Q26
Trust	3	5	Q13, Q47
Dementia	5	7	Q7
Support	2	8	Q17, Q42, Q43
Safety	3	9	Q14, Q27, Q28, Q50, Q56
efficiency of vaccine	4	12	Q38, Q39
Misinformation	5	15	Q38, Q39
back to normal	6	16	Q6, Q19
Impact of relatives	6	19	Q12, Q14
Negative staff	4	15	Q24, Q25
Age	6	16	Q31
Social media	6	21	Q34, Q35, Q36, Q37, Q38, Q39, Q40, Q41, Q42, Q43
Side effect	7	28	Q23, Q30, Q51
Control of information	7	28	Q10, Q27, Q28, Q40, Q41, Q36, Q37, Q34, Q35, Q38, Q39, Q54, Q57, Q58, Q59
difficulties	6	53	Q20, Q18, Q21, Q26, Q29, Q48, Q49, Q53
Fear	5	49	Q51, Q23, Q30
Encouragement	7	59	Q9, Q17, Q22, Q55
Programme	7	73	Q8, Q45, Q46, Q47, Q50, Q55, Q60

Table S2 Questions included in testing H_1, H_2, and H_3 hypotheses

	**Motivation**	**Social media (H_1)**	**Safety of the vaccine (H_2)**	**Programme and Process questions (H_3)**
**Residents**	Q6, Q8, Q9, Q12, Q13	Q34, Q37, Q39, Q40	Q14	Q7, Q10, Q11, Q18, Q20, Q21, Q26, Q29, Q42, Q45, Q46, Q47, Q48, Q49, Q50, Q51*, Q52, Q53*, Q54, Q55, Q56, Q57, Q58, Q59, Q60
**Staff**	Q17, Q19, Q22, Q24*, Q25	Q35, Q36, Q38, Q41, Q43	Q23, Q27, Q28, Q31	

** Those questions (Q24, Q51, and Q53) were hypothesized to be relevant but factor analysis found that they were not consistent to the construct, thus, were omitted.*

Table S3: Codes and frequencies for other factors that increased the uptake of vaccine by residents in care homes (Q15 in the questionnaire)

Code	Respondent Number	Quotation*	Frequency
COVID outbreak	2	previous outbreak of COVID within the home.	1
Size of the care home	3	We were not part of vaccine programme because we are a 4 bed unit	1
Back to normal	4	the hope of being physically closer to a loved one	7
	23	residents wanted to return to a normal life	
	24	To allow visitors back into the home	
	42	The desire to be reunited with their families through visitation and day trips.	
	46	residents wanted to go back to normal	
	53	Restrictions on visiting	
	59	Families hopeful that visiting would resume to a normal level	
GPs support	12	GP - Instruction and Directed	2
	35	GP support	
Communication	14	Good communication between Residents, Relatives and Staff	4
	30	easy reads - communication, visually seeing others take the vaccine helped	
	40	Strong support/relations/trust between residents and care home manager/staff	
	48	Staff support	
Risk of contracting the virus	25	02 therapy in the event of getting Covid was a major factor in the uptake.	3
	34	RESIDENTS WERE VERY KEEN TO RECEIVE THE VACCINE - TO TRY AND LOWER THEIR RISK	
	63	Fear of having covid-19 as a lot of residents had been watching the news stories on the tv	
Vaccination team	29	Efficiency of vaccination team	3
	50	Residents who did not get their vaccination on the days that the Trust came to the Home were very keen to get the vaccination as soon as they were able after that.	
	58	The ability to receive it quickly after admission to the care home.	
Trust in staff	52	The fact that staff were also getting it was a reassurance to residents and their families	3
	54	The staff getting it helped	
	55	re assured that staff are on duty 24 hours per day if they were feeling unwell.	
TV	42	Watching other elderly people getting the vaccine on the television.	1
Family	43	families predominately	2
	59	Families hopeful that visiting would resume to a normal level	

*Recorded as it is without corrections

Table S4 Codes and frequencies for other factors that decreased the uptake of vaccine by residents care homes (Q16 in the questionnaire)

Code	Respondent Number	Quotation *	Frequency
Uncontrolled	4	religious views and beliefs in one situation	1
admissions/discharges	6	high volume of admissions/discharges	2
	36	As it is a new home opened, residents are having to be re registered, which has held up vaccinations, We had offered one resident the vaccine who had just moved into facility, but he declined, and refused to take the injection.	
Behaviour	12	behavioural issue	1
Trust in the vaccine	15	new and untested and would not give consent for parents who are both residents to have vaccine.	3
	25	Residents consent and allergies	
	46	All the residents have been vaccinated apart from one resident who was agitated and refused to be vaccinated	
Do not believe in vaccine	23	never availed of any vaccine	2
	48	Only those residents who would have refused the Influenza Vaccine in the past.	
Side effects	25	Residents consent and allergies	3
	38	fear of side effects	
	66	previous allergies	
GP delays	29	Delay by GP's to come to the home to vaccinate residents who hadn't been vaccinated by vaccination team.	3
	35	Lack of GP support	
	42	Uncooperative GP in the case of one resident who has no capacity to give consent	
Needs	30	The residents complex needs made it more difficult	1
Vaccinator	40	Some vaccinators had limited understanding in how to approach/manage challenging residents	1
Unwell	43	only if someone was unwell	1
Family	31	family feud	2
	58	A few families were apprehensive	
Consent	52	Mainly the paperwork for consents, which is always off putting for any activity	1

*Recorded as it is without corrections

Table S5 Codes and frequencies for other factors that increased the uptake of vaccine by staff (Q32 in the questionnaire)

Code	Respondent Number	Quotation*	Frequency
Back to normal	2	the ability to have some normality back in life	14
	7	Trust in management and the desire for life to get back to normal	
	16	First round of vaccines small intake. As barriers or concerns re travel were noted then vaccine uptake increased	
	17	The hope of ending lockdown and restrictions. Being able to meet with family who live outside the UK	
	11	feeling they would be able to go abroad	
	22	just fear of the unknown	
	23	return to normal life	
	25	the idea of needing a vaccine to go on holiday, pub etc	
	42	The hope that there would be a relaxation of the weekly Covid testing and wearing of PPE at all times if you were vaccinated which turned out to be untrue.	
	45	the fact that they thought they could travel abroad if they had the vaccine.	
	46	Staff wanted to go back to normality	
	63	The belief that they would be unable to travel abroad without being vaccinated	
	66	they were concerned they wouldn't be able to go on holiday	
	57	MOST STAFF WERE HAPPY TO TAKE IT AS THEY THOUGHT THINGS WOULD GO BACK TO NORMAL AFTER VACCINATION	
Happy to take it	3	All staff were happy to take it	4
	29	Staff became keener to have vaccine as time passed	
	30	Some staff took the vaccine later than others while they waited to see how others reacted	
	57	MOST STAFF WERE HAPPY TO TAKE IT AS THEY THOUGHT THINGS WOULD GO BACK TO NORMAL AFTER VACCINATION	
Protection	4	yes, the potential of protection for our residents and themselves, as a team we managed a challenging outbreak and never ever want to go through such an awful experience again.	3
	47	wanting protection	
	55	Prevention of covid or less likely to take it.	
Outbreaks	4	yes, the potential of protection for our residents and themselves, as a team we managed a challenging outbreak and never ever want to go through such an awful experience again.	3
	5	An active outbreak in Jan which resulted in death of one resident frightened staff	
	22	just fear of the unknown	

Encouragement and communication	6	ongoing encouragement by management and community stats	11
	12	Encouragement and Correct information to make a informed decision	
	14	Conversations between Staff and Home Manager	
	15	Encouragement by Management. Seen as for the greater good of our Residents	
	32	i GAVE REGULAR UPDATES TO STAFF ON ALL INFORMATION	
	58	Positive engagement between staff and vaccinators. They could help ease worries around the vaccination	
	59	examples by management and their peers	
	50	Home Manager rang each staff member individually and encouraged staff to have the vaccine and answered any queries. >80% up take of vaccine by staff to date	
	52	All getting it together here in the Home on the same day - we created a bit of an atmosphere and offered staff breakfast while they were waiting before/after.	
	7	Trust in management and the desire for life to get back to normal	
	54	The manager taking it	
Trust	7	Trust in management and the desire for life to get back to normal	1
Part of the job	11	The short notice period given, feeling they would be able to go abroad, not being informed that it was different method of vaccination that what they were used to previously, have CV individuals in their household, they felt they had to or it would be frowned upon, the possibility of losing their jobs	5
	34	STAFF FELT THEY HAD A MORAL AND PROFESSIONAL RESPONSIBILITY TO RECEIVE THE VACCINE	
	42	The hope that there would be a relaxation of the weekly Covid testing and wearing of PPE at all times if you were vaccinated which turned out to be untrue.	
	43	vaccination provided in the workplace	
	52	All getting it together here in the Home on the same day - we created a bit of an atmosphere and offered staff breakfast while they were waiting before/after.	
Follow colleagues	18	Once some staff had taken the 1st dose others than followed	1

*Recorded as it is without corrections

Table S6 Codes and frequencies for other factors that decreased the uptake of vaccine by staff (Q33 in the questionnaire)

Code	Res	Quotation*	Frequency
Peer pressure	2	Peer pressure from colleagues, fear of side effects	3
	28	Some staff waited to see if there were any side effects following second vaccination before going themselves	
	43	some peer pressure	
Side effects	2	Peer pressure from colleagues, fear of side effects	12
	3	there was concern re: side effects and working the next day	
	17	Pregnancy complication - Severe allergy history - Fear of new vaccine- Risk complications	
	26	Side effects in future	
	28	Some staff waited to see if there were any side effects following second vaccination before going themselves	
	30	Lack of evidence on the long term effects from taking the vaccine	
	42	Fears around effects on female fertility. Staff from countries where they allege vaccinations were administered to infect the population with incurable diseases. This was mostly based on fake news.	
	46	suspected side effects	
	50	Potential side effects and safety concerns	
	53	Young staff heard about fertility issues staff pregnant and with allergies	
	55	Some staff heard that there were side effects and this prevented them taking it.	
	63	Fear of long term side effects of the vaccine	
Social media	4	social media scare mongering, not enough action was taken to prevent this in the media	5
	5	Social media/sensationalism regarding conspiracy theories etc	
	27	Social media platforms	
	47	Social media	
	59	Social media	
Pregnancy and gender	6	pregnancy issues	15
	16	Females who were considering having children in the future refused the vaccine	
	17	Pregnancy complication - Severe allergy history - Fear of new vaccine- Risk complications	
	11	Younger staff followed the PHA advice at the time regarding pregnancy or planning pregnancy, the speed of the roll out, previously having covid and having anti-bodies, the emergency approval element, no long term data, censorship of other professionals with their findings. hypothesis, inadequate 'debunking' of other professional opinion, one vaccine being linked to blood clots and then ceased for certain age groups, the adverse reactions by other staff, residents and relatives, the data on the yellow card reporting system along with years, other visiting	

		professionals to the home advising they were not taking it, a comment by a GP following the sudden death of a resident, no coercion in the care home	
	18	Young female staff were concerned this would affect their fertility	
	23	a lot of under 40 female staff refused vaccine	
	25	fear it affected fertility	
	34	YOUNGER STAFF WERE RELUCTANT DUE TO RISK RE FERTILITY CONCERNS	
	40	Vaccine was rolled out in our home in December, in the height of all the negativity, uncertainty and limited information and conspiracy around affecting fertility, this played a massive part in lower uptake	
	42	Fears around effects on female fertility. Staff from countries where they allege vaccinations were administered to infect the population with incurable diseases. This was mostly based on fake news.	
	52	Younger staff and the initial ambiguity around impact for those planning on being pregnant.	
	53	Young staff heard about fertility issues staff pregnant and with allergies	
	57	UNCLEAR INFOMRATION IN LITERSTURE SUPPLIED TO STAFF I.E REGARDING FERTILITY AND LITERATURE DID NOT HAVE ASSURANCES RE EFFECTIVENESS OF VACCINE OR HOW LONG IT WOULD LAST	
	60	Female staff wanting to start a family	
	66	some staff were worried about fertility rates	
Age	11	Younger staff followed the PHA advice at the time regarding pregnancy or planning pregnancy, the speed of the roll out, previously having covid and having anti-bodies, the emergency approval element, no long term data, censorship of other professionals with their findings. hypothesis, inadequate 'debunking' of other professional opinion, one vaccine being linked to blood clots and then ceased for certain age groups, the adverse reactions by other staff, residents and relatives, the data on the yellow card reporting system along with years, other visiting professionals to the home advising they were not taking it, a comment by a GP following the sudden death of a resident, no coercion in the care home	5
	18	Young female staff were concerned this would affect their fertility	
	23	a lot of under 40 female staff refused vaccine	
	34	YOUNGER STAFF WERE RELUCTANT DUE TO RISK RE FERTILITY CONCERNS	
	45	staff said they don't trust the vaccine, this was young staff.	
beliefs and culture	10	beliefs and culture	2
	15	Religious / cultural reasons	
Contracting the virus	11	Younger staff followed the PHA advice at the time regarding pregnancy or planning pregnancy, the speed of the roll out, previously having covid and having anti-bodies, the emergency approval element, no long term data, censorship of other professionals with their findings. hypothesis, inadequate 'debunking' of other professional opinion, one vaccine being linked to blood clots and then ceased for certain age groups, the adverse reactions by other staff, residents and relatives, the data on the yellow card reporting system along with years, other visiting	1

		professionals to the home advising they were not taking it, a comment by a GP following the sudden death of a resident, no coercion in the care home	
Trust in the vaccine	11	Younger staff followed the PHA advice at the time regarding pregnancy or planning pregnancy, the speed of the roll out, previously having covid and having anti-bodies, the emergency approval element, no long term data, censorship of other professionals with their findings. hypothesis, inadequate 'debunking' of other professional opinion, one vaccine being linked to blood clots and then ceased for certain age groups, the adverse reactions by other staff, residents and relatives, the data on the yellow card reporting system along with years, other visiting professionals to the home advising they were not taking it, a comment by a GP following the sudden death of a resident, no coercion in the care home	8
	12	Does it believe in efficacy of the vaccine due to the quick development and does not believe of the virus	
	29	Alerts re conception and clots have slowed off vaccine uptake	
	30	Lack of evidence on the long term effects from taking the vaccine	
	31	So many confusing information in different platforms	
	40	Vaccine was rolled out in our home in December, in the height of all the negativity, uncertainty and limited information and conspiracy around affecting fertility, this played a massive part in lower uptake	
	57	UNCLEAR INFOMRATION IN LITERSTURE SUPPLIED TO STAFF I.E REGARDING FERTILITY AND LITERATURE DID NOT HAVE ASSURANCES RE EFFECTIVENESS OF VACCINE OR HOW LONG IT WOULD LAST	
	58	Lots of mis-information regarding the vaccine's benefits or safety	

*Recorded as it is without corrections

Table S7 Codes and frequencies for approaches that would be helpful moving forward in relation to using and managing social media (Q44 in the questionnaire)

Code	Respondent Number	Quotation*	Frequency
Information	2	More accurate information available regarding the vaccines instead of negative press	10
	4	a holistic approach would be better, too many people see what's happening in England, Scotland Wales Ireland and NI and the differences and this becomes very confusing. Better law to remove false information and a quicker approach to dispelling myths	
	12	Social Media Campaign should be introduces weeks before the vaccination started and to include benefits ,should be easily understood ,more pictures or animation rather than medical terms that is not easily understood by the general public	
	5	Social media is beneficial but there is so much in the way of misinformation which leads to negative impact particularly in relation to the pandemic/ vaccination programme. I did use information obtained from across social media platforms (from PHA, RCV for example) to help encourage staff to come forward for vaccination. Lots of reputable information from established organisations necessary to help dispel any myths	
	24	More information provided to care home promptly	
	30	Give one message	
	31	Information should be release to the HSC and independent sector first, before press release. By the time it reaches the HSC and private sector, the regulation/guidelines was already changed and the credibility of the HSC, care or residential home following through the regulation/guidelines is being questioned.	
	40	More factual information. Discredit the conspiracy facts with strong scientific evidence, focus on disputing all the concerns as a main point.	
	47	clear constant messaging about reading information from trusted sources	
	50	Difficulty in ensuring that information on social media is Accurate and beneficial for staff.	
Law	4	a holistic approach would be better, too many people see what's happening in Ireland, Wales, and NI and the differences and this becomes very confusing. Better law to remove false information and a quicker approach to dispelling myths	3
	16	Remove negative vibes and scare mongering	
	31	Information should be release to the HSC and independent sector first, before press release. By the time it reaches the HSC and private sector, the regulation/guidelines was already changed and the credibility of the HSC, care or residential home following through the regulation/guidelines is being questioned.	
Encouragement	5	Social media is beneficial but there is so much in the way of misinformation which leads to negative impact particularly in relation to the pandemic/ vaccination programme. I did use information obtained from across social media platforms (from PHA, RCV for example) to help encourage staff to come forward for vaccination. Lots of reputable information from established organisations necessary to help dispel any myths	3

	53	Good news stories not told	
	57	FOR EVERY POSITIVE STORY THERE SEEMS TO BE MORE NEGATIVE. PEOPLE GIVING DEFINITE ANSWERS TO PEOPLES CONCERNS WOULD BE REASSURING	
Positive platform	10	social media should have showed more of the positive outcome of the vaccine	5
	18	A video in layman's terms of who and why the vaccines is safe and works to show staff	
	42	Medical Consultants, Doctors Nurses and Scientist should/could have supported the vaccination campaign more actively on Social media. Fake News MUST be reported and the source prosecuted.	
	46	Social media should be more transparent	
	57	FOR EVERY POSITIVE STORY THERE SEEMS TO BE MORE NEGATIVE. PEOPLE GIVING DEFINITE ANSWERS TO PEOPLES CONCERNS WOULD BE REASSURING	
Negative platform	11	Social media is a barrier to informed consent	3
	52	I do not believe it was helpful at all - even positive campaigns were followed by a litany of negative comments that were read by all who were in any doubt.	
	58	The social media message will probably get drowned out by the plethora of misinformation. This means the message fails to get the required traction. Tackle misinformation and use traditional advertisement to get the message across.	

*Recorded as it is without corrections

Figure S1: An illustration of the codes from seven semi structured interviews

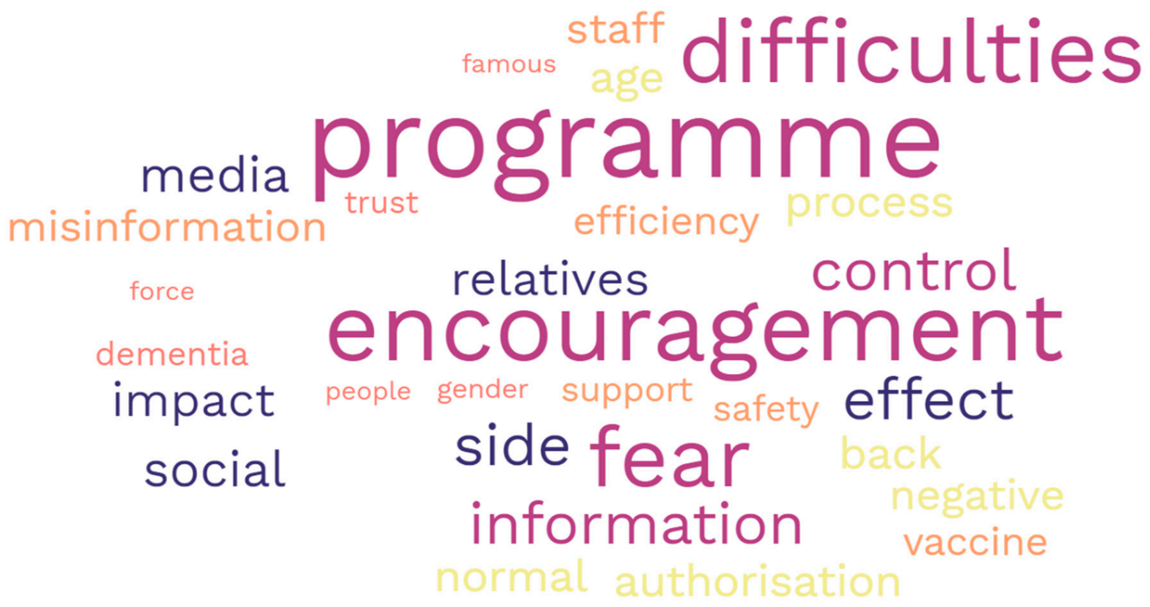

Supplement: Supplementary file 1 [file vaccines-09-01160-s001.zip › vaccines-1396822-supplementary.pdf]
